# Urine and bladder washing cytology for detection of urothelial carcinoma: standard test with new possibilities

**DOI:** 10.2478/v10019-010-0042-8

**Published:** 2010-09-29

**Authors:** Margareta Strojan Flezar

**Affiliations:** Institute of Pathology, Faculty of Medicine, University of Ljubljana, Ljubljana, Slovenia

**Keywords:** cytology, urine, bladder washing, urothelial carcinoma

## Abstract

**Background:**

Light microscopic evaluation of cell morphology in preparations from urine or bladder washing containing exfoliated cells is a standard and primary method for the detection of bladder cancer and also malignancy from other parts of the urinary tract. The cytopathologic examination is a valuable method to detect an early recurrence of malignancy or new primary carcinoma during the follow-up of patients after the treatment of bladder cancer.

**Conclusions:**

Characteristic cellular and nuclear signs of malignancy indicate invasive or *in situ* urothelial carcinoma or high-grade papillary urothelial carcinoma. However, low sensitivity of the method reflects the unreliable cytopathologic diagnosis of low-grade urothelial neoplasms as cellular and nuclear signs of malignancy in these neoplasms are poorly manifested. Many different markers were developed to improve the diagnosis of bladder carcinoma on urinary samples. UroVysion™ test is among the newest and most promising tests. By the method of *in situ* hybridization one can detect specific cytogenetic changes of urothelial carcinoma.

## Introduction

The examination of urine is one of the oldest medical procedures dating back to the Old Egypt.^1^,^2^ First microscopical examination of the cells in the urinary sediment was reported by the Czech doctor Lambl back in 1856.^2^

At present the cytopathological examination of urine or other fluid samples from the urinary tract is a routine noninvasive diagnostic procedure to detect cancer of the urinary tract, foremost bladder cancer especially in patients with painless haematuria.^3^,^4^ It is also used during the follow-up procedures of the patients previously treated for bladder cancer in order to early detect recurrence or new primary.^4^ Exceptionally, the cytopathological examination of urine is used for the screening for urothelial carcinoma in the high risk population.

The cytopathological examination is a highly specific method for the diagnosis of invasive and *in situ* urothelial carcinoma and high-grade papillary carcinoma, however it is notorious of being unreliable for the detection of low-grade papillary neoplasms.^5^,^6^

## Preparation of fluid samples from the urinary tract for cytopathological examination

A most common sample from the urinary tract is spontaneous – voided urine. Bladder washing samples are also very frequent samples sent to the cytology laboratory. Other samples such as catheterized urine or urine obtained by the retrograde catheterization of urethers or renal pelvis are sent for the cytopathological examination only occasionally.

The second morning voided urine is the most appropriate sample for the cytopathological examination as it contains enough of preserved cells. The first morning urine contains more cells but they show different degrees of degeneration being exposed to the acid milieu of urine through the night and are less suitable for the cytological evaluation. Because the cells exfoliate from the urothelium intermittently, three urine samples should be examined from three consecutive days to ensure that diagnostic cells were sampled.^2^

The bladder washing sample is obtained during or prior cystoscopy which is an invasive diagnostic procedure for the macroscopical evaluation of the bladder mucosa. First the bladder should be emptied by a catheter. Then 50 to 100 ml of normal saline is instilled and recovered and this procedure is repeated three times.^2^ Bladder washing exfoliates large sheets of urothelium and even three-dimensional urothelial fragments. Therefore, bladder washing samples are highly cellular and contain well preserved cells.

When fluid samples cannot be delivered to the cytology laboratory within three hours after they were obtained, they can be prefixed with a mixture of 2% polyethylen glycol (Carbowax™) and 50% to 70% ethanol.

Different techniques are used for the cytopathological preparation of fluid samples of the urinary tract. Some laboratories still use a centrifugation of fluid and then the pellet is directly smeared onto the glass slide. Other laboratories introduced the commercial ThinPrep™ technique for the preparation of samples from the urinary tract.^7^ ThinPrep™ was first developed for the preparation of cervical cytology samples. The membrane filtration technique is used in several laboratories including ours. Urine or bladder washing sample is filtered through the polycarbonate membrane filter with 5 μm pores (Costar® filter system, Costar Europe Ltd., Netherlands, Europe; Nucleopore® filter, diameter 47 mm, pores 5 μm, Whatman Inc., New Jersey, USA), so predominantly urothelial cells remain on the filter. Usually the majority of erytrocytes and leukocytes are removed because the gentle negative pressure is applied to assist filtration, which deforms these cells so they pass through the filter. The cell monolayers are obtained by gently imprinting filter onto a pair of glass slides. The cell sample on the slide should be fixed by the immediate immersion into Delaunay fixative (acetone: 96% ethanol 1:1 + 0.5 ml/l trichoracetic acid) or fixed by spraying with Merckofix® (Merck KGaA, Darmstadt, Germany). Cell preparations are subsequently stained by the Papanicolaou method.

## Cytopathological diagnosis of urothelial tumours

The last WHO classification of the tumours of the urinary system (published in 2004) divides urothelial neoplasms into infiltrating (invasive) urothelial carcinomas and non-invasive urothelial carcinomas.^8^,^9^ Later they are further subdivided into low and high-grade papillary carcinomas, papillary urothelial neoplasms of low malignant potential (PUNLMP) and papillomas on one side and urothelial carcinomas *in situ* on the other side.^9^

In his last edition of Diagnostic cytology and its histopathologic bases, Koss suggested that for the purpose of cytopathological evaluation the urothelial carcinomas should be divided into papillary and non-papillary carcinomas.^5^ The reason is that cytopathological diagnosis of non-papillary carcinomas, including invasive and *in situ* carcinomas is very reliable (specificity ranging from 88.1 to 99.%, mean 97.1%; our data 96%, Table 1), while the cytopathological evaluation of papillary neoplasms which are often of low-grade is notorious for being of limited usefulness.^5^,^10^–^12^

Another obstacle of the cytopathological evaluation is that the true origin of malignant cells found in urine cannot be reliably identified. Malignant cells found in urine can originate not only from bladder, but from any part of the urinary tract, namely from renal pelvis, urether or urethra.

### Infiltrating (invasive) urothelial carcinoma

**FIGURE 1.**
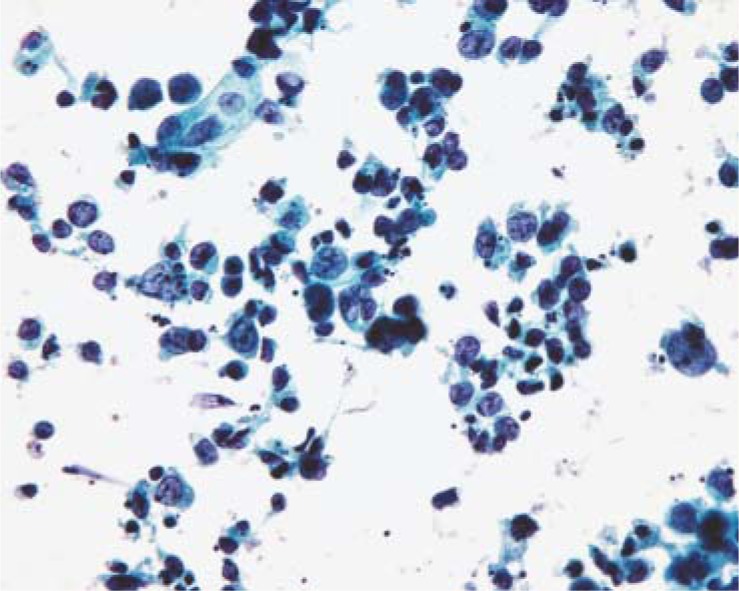
Malignant cells of invasive urothelial carcinoma with cellular debris (necrosis) in the background (Papanicolaou, ×400).

Among non-papillary carcinomas the cells of invasive urothelial carcinomas usually exhibit clear cytological and nuclear characteristics of malignancy in voided urine or bladder washing samples.^5^,^6^,^10^ Specifically, polymorphous cells with increased nuclear-cytoplasmic ratio, polymorphous nuclei, nuclear hyperchromasia with coarsely granular and unevenly distributed chromatin, and nucleoli are observed (Figure 1). The cellularity of samples partially depends on the type of specimen, namely larger number of malignant cells is found in bladder washing, while the cell degeneration with pyknosis is more pronounced in voided urine samples. Cells lay singly or in poorly cohesive clusters. Background may contain necrotic debris, blood and inflammatory cells. Sensitivity of cytology for the detection of invasive urothelial carcinoma is high (81–100%, our data: 100%, Table 1).^5^,^10^,^11^

### Urothelial *in situ* carcinoma

**FIGURE 2.**
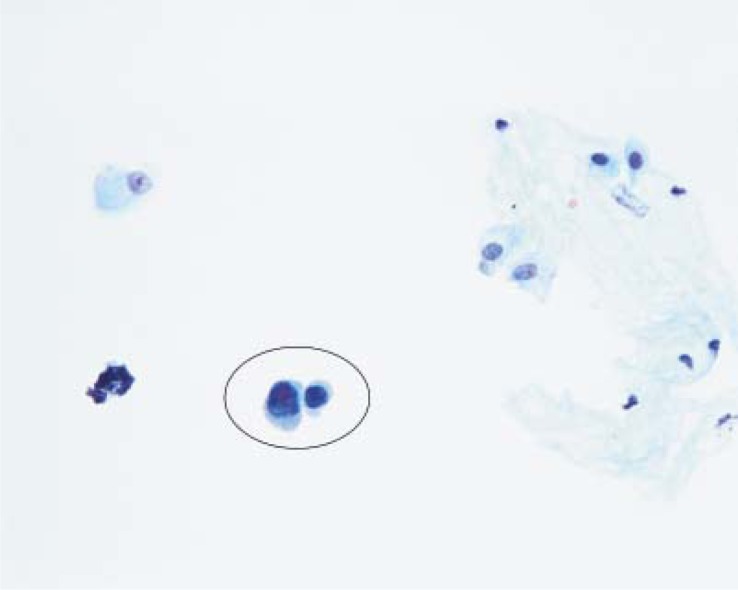
Two malignant cells (in circle) of in situ urothelial carcinoma (note: clear background) (Papanicolaou, ×400).

Also the urothelial *in situ* carcinoma exfoliates cells with evident malignant morphology, similar to cells of invasive urothelial carcinoma (Figure 2).^5^,^6^ In voided urine samples the cells are of an intermediate size or small, mostly laying singly. Single bizarre cells can be observed. Nuclei are large, of irregular shape, hyperchromatic, contain coarse chromatin, large nucleoli; pyknosis is present frequently. Cytoplasm is scanty. In contrast to invasive urothelial carcinoma, generally no necrosis, scanty erythrocytes or leukocytes are found in the background of samples containing cells of urothelial in situ carcinoma. Due to the obvious morphological signs of malignancy the sensitivity of cytology for the detection of urothelial in situ carcinoma is high (70–100%; our data: 100%, Table 1).^5^,^10^,^11^ However, it is difficult to tell apart reliably the malignant cells of the *in situ* carcinoma from the cells of invasive carcinoma even when the characteristics of the background are considered in the cytopathological diagnosis.

### High-grade papillary urothelial carcinomas

Among papillary tumours, high-grade papillary urothelial carcinomas (including former WHO classification grade II and III) shed cells with cytological atypia consistent with malignancy, as described above. The majority of high-grade carcinomas of former grade III exfoliate evident malignant cells, while in 20–30% of former grade II carcinomas the cytological atypia is less pronounced. Sensitivity of cytology for the detection of high-grade papillary urothelial carcinomas of former grade II and III combined is 72%, however for papillary urothelial carcinomas of former grade III is 91% (our data 94%, Table 1).^5^,^10^,^11^

### Low-grade papillary urothelial carcinomas and other low-grade papillary neoplasms

**FIGURE 3.**
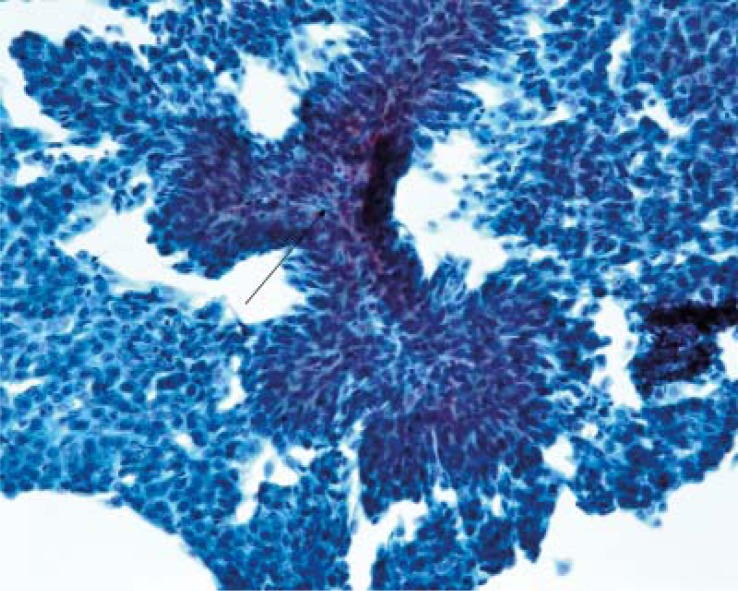
Papillary structure covered with mildly atypical urothelial cells diagnostic of low-grade papillary urothelial neoplasm (Papanicolaou, ×400).

On the contrary, low-grade papillary urothelial carcinomas are difficult to diagnose in cell samples, because the cytological signs of malignancy are not obvious.^5^,^6^ Cells and nuclei are rather uniform, nuclear-cytoplasmic ratio is not obviously increased. Nuclei are only slightly or moderately enlarged, chromatin is relatively bland. These nuclei are difficult to recognize as malignant in cytology. The background is typically clean, some erythrocytes can be found. Only rarely true papillary fragments containing fibrovascular core can be found, but are not specific for papillary carcinomas; they could belong to PUNLMP or papillomas (Figure 3). Urinary cytology is not reliable for diagnosing low-grade papillary carcinoma and other low-grade papillary neoplasms. Sensitivity for the detection of low-grade papillary tumours is low, however various percentages are reported in the literature ranging from 0–73% (majority between 30 to 40%; our data: sensitivity 18% for the positive diagnosis and 55% for the combined positive/suspicious diagnosis, Table 1).^5^,^10^–^13^

### Differential diagnosis of inconclusive cytological atypia

**FIGURE 4.**
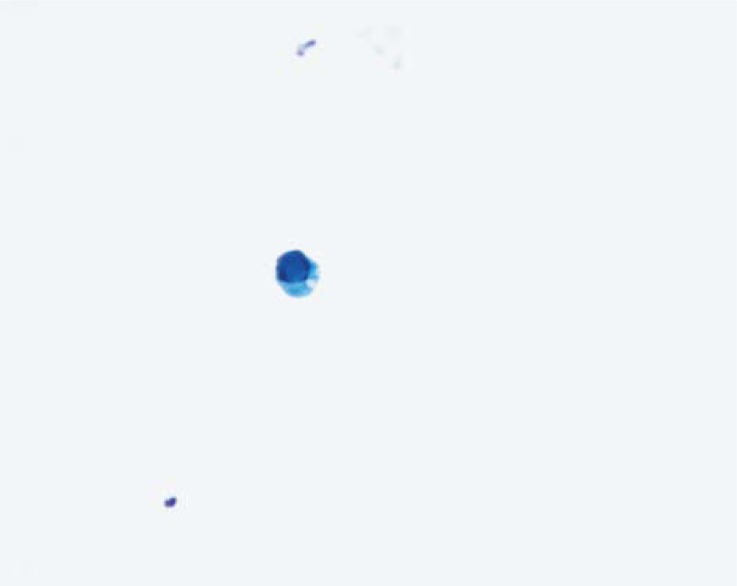
Typical polioma virus cytopathic effect on urothelial cell (Papanicolaou, ×400).

In low-grade papillary urothelial carcinomas and other low-grade papillary neoplasms cells exhibit some degree of cytological atypia described above. However, several benign lesions can show similar cytological atypia, namely reactive atypia related to inflammation, stones in the urinary tract or instrumentation.^5^,^6^ Also the post-treatment reactive urothelial changes could be pronounced and have to be taken into consideration. Cytological atypia of reactive type can be very prominent after the irradiation of bladder, intravesical chemotherapy with mitomycin or immunotherapy with Bacillus Calmette-Guérin (BCG) (used for the therapy of carcinoma *in situ*). The polyoma virus infection produces the so called decoy cells with enlarged, usually round nuclei that have typical intranuclear viral inclusions (Figure 4). The chromatin has appearance of ground glass, with condensation of chromatin at the nuclear border, so called type 1 nuclear changes. The cytoplasm of decoy cells is scarce to moderate, thickened or degenerated, may have a comedo shape. Other three types of polyoma related cytological changes are described but are not so reliably recognized in routine setting.^2^

### Non-urothelial carcinomas of the urinary tract

In rare instances also non-urothelial malignant cells are observed and can be diagnosed by the cytopathological examination of cell samples from the urinary tract. The most common non-urothelial carcinoma is squamous cell carcinoma.^5^ It can exfoliate cells with obvious squamous features, namely orangeophylic cytoplasm that is well demonstrated in Papanicolaou stained cell preparations. When combined with malignant cytological features the diagnosis of squamous cell carcinoma can be made on urine or bladder washing sample. However, it is difficult if not impossible to differentiate whether malignant squamous cells originate from squamous cell carcinoma of bladder or they belong to the part of urothelial carcinoma of bladder with squamous differentiation. One also has to bear in mind that in the urinary samples from female patients the malignant squamous cells could originate from squamous cell carcinoma of the uterine cervix with exfoliated cells in the vaginal excretions washed by urine or by direct invasion of squamous cell cervical carcinoma into the bladder.

In males, adenocarcinoma of the prostate can exfoliate cells into the urine, occasionally they are found in bladder washings.^5^ Roundish glandular like structures of malignant cells can be found, with the cytological atypia roughly reflecting the grade of prostate adenocarcinoma. Immunocytochemical staining with antibody to prostate specific antigen (PSA) can confirm the final diagnosis of prostatic adenocarcinoma.

## Ancillary urine-based techniques for the diagnosis of urothelial bladder cancer

Although the cytopathological examination of urine or bladder washing cell samples is very specific (97%; our data: 96%, Table 1) it suffers from low sensitivity especially in the case of low-grade papillary tumours.^5^,^10^–^13^ This type of tumours is prone to recurrence and it is found in 70% of patients, furthermore 5% of them develop invasive carcinoma.^13^ A specific clinical problem are patients with early invasion into *lamina propria* at first diagnosis. In these patients the disease progresses to the muscular invasive form in 20–30% of cases and the progression potentially leads to a fatal outcome.^13^ The patients treated previously for urothelial carcinoma are therefore followed-up regularly with cystoscopy and cytology. Due to the above mentioned limitations of cytology the need for new non-invasive techniques to detect recurrences has emerged.^13^ However, although the new markers exhibit better sensitivity than cytology only few could reach the high specificity of cytology.

### DNA ploidy

In the seventies and eighties of the last century the researchers and pathologists were using DNA cytometry to measure DNA ploidy of urothelial tumors.^14^,^15^

There was found that the non-invasive low-grade urothelial tumours were predominantly diploid, while grade II urothelial carcinomas were diploid in about 50% of cases while the other 50% were aneuploid. The grade III tumours and carcinomas *in situ* were predominantly aneuploid. When correlating the DNA ploidy to clinical data they found that aneuploid tumours were associated with tumour persistence, recurrence, and progression to invasion.^16^ However, DNA diploidy in low-grade tumours could not improve the prediction of recurrence which is very frequent in these tumours. DNA ploidy measurement in urothelial tumours has reached its limitations, so new ancillary methods were searched for.

### ImmunoCyt/uCyt™

This cytology based test was developed in 1997. It is an immunofluorescence based test, using three monoclonal antibodies, two of them (M344 and LDQ10, labelled with fluorescein green) are directed against mucin-like antigens related to urothelial carcinoma.^17^,^18^ They were found to be positive in 71% of non-invasive (pTa) or early invasive (pT1) tumours. The third antibody (19A211 labelled with Texas red) is directed against high molecular weight carcinoembrionic antigen (CEA). It was found to be positive in 90% of non-invasive (pTa) or early invasive (pT1) tumours. Sensitivity of the test was shown to be 53–100% (mean 90%) also for low-grade tumours, while the specificity was 64–95% (74%), which is less than cytology. The test obtained FDA clearance in 2000 for the detection of malignant cells in urine in patients treated for urothelial cancer.

### BTA stat^®^

Bard BTA stat^®^ (bladder tumour antigen test)^®^ (Polymedco, Cortland Manor, NY, USA) is a soluble urine marker test that was aimed at the basal membrane antigen detection (complement factor H-related protein) in the urine using latex agglutination test (immunoassay).^13^ The test showed variable sensitivity (34%–100%) and especially its sensitivity for low-grade tumours was rather modest, while the specificity was in the same range (40–96%). However, the high false positive rate (4–34%) makes the test debatable for a wider clinical use. FDA approved the test to detect bladder cancer in voided urine.

### NMP22 (nuclear matrix protein)™ immunoassay

NMP22™ test (Matritech, Newton, MA, USA) is a soluble urine marker test. NMP22 (nuclear matrix protein) is a member of family of nuclear matrix proteins that are involved in DNA configuration, structure and function.^13^,^19^,^20^ It was shown that the sufficient difference existed between normal and urothelial cancer cells to be used as a diagnostic test. The NMP22™ detection method is an immunoassay that showed high sensitivity (60–86%) for the detection of urothelial neoplasia, however the specificity is bellow that of cytology (48–81%) producing many false positive tests. Besides, the test was reported to be rather inconvenient and costly. Anyhow, the FDA approved to detect bladder cancer in voided urine, adjunct to cystoscopy.

### Other potential urinary markers of urothelial carcinoma

Many other markers either cell based (microsatellite analysis, telomerase detection, Quanticyt nuclear karyometry) or soluble urine markers (BLCA-4, BLCA-1, HA-HAse, survivin) were reported to be useful for the detection of urothelial cancer.^13^ The majority exhibited higher sensitivity than cytology, however they didn’t reach the high specificity of cytology and did not obtain the FDA approval for the clinical use.

## Multitarget multicolour fluorescence *in situ* hybridization (FISH) UroVysion™ test

High frequency of specific chromosomal abnormalities in urothelial cancers was found in the nineties and several DNA probes were made to detect these abnormalities.^21^,^22^ Initial studies tested single DNA probes using FISH for the detection of urothelial carcinoma, however single probes resulted in limited specificity and sensitivity. The procedures were also time consuming, therefore they could be not introduced into the routine clinical management of the patients.

The study of Sokolova *et al.* showed that the application of several DNA probes combined significantly increased the sensitivity for the detection of abnormal cells.^23^ In their study they tested ten FISH probes and found that the highest sensitivity was achieved using three chromosome enumeration probes (CEP), namely for chromosome 3 (labelled by Spectrum red), chromosome 7 (labelled by Spectrum green), chromosome 17 (labelled by Spectrum aqua) and one locus-specific identifier (LSI) probe for 9p21 (labelled by Spectrum gold). In their study the cut-off value set at 5 abnormal cells yielded sensitivity 84%, specificity 92% for the detection of urothelial carcinoma. Based on their observation the commercially available multicolour multitarget FISH UroVysion™ test (Abbott Molecul Inc., Des Plaines, IL, USA) incorporating all four DNA probes was made.^24^ Initially it was FDA approved in 2001 for the surveillance of patients with bladder cancer, later it was approved also for the detection of bladder cancer in persons with haematuria suspected of having bladder cancer. In other words, UroVysion™ can be used for screening of bladder cancer in patients with haematuria.

Already in 2002 the studies using commercial UroVysion™ test were published. One of the first was the study by Bubendorf *et al*. who showed that UroVysion™ could facilitate the diagnosis of bladder cancer and detect the recurrence.^25^ They claimed that the test was a rapid, simple and powerful diagnostic method. Either voided urine or bladder washing samples prepared as cytospins could be used. They found that the sensitivity for the detection of non-invasive carcinoma was 73%, while later studies showed sensitivity ranging from 36–86%. The sensitivity for the detection of invasive carcinoma was even higher reaching 100%, and other studies confirmed 94–100% sensitivity. The specificity in their study was 96%, in the later studies up to 100%.^26^–^29^ They suggested that the cystoscopy examination should follow a positive test even in the absence of suspicious or positive cytology. Although the test is rather expensive, the cost benefit ratio was supposedly lower taking into account the decreased need for the diagnostic cystoscopy.

In one of the later studies Yoder *et al.* suggested that if cytology was positive and used as the first diagnostic test no UroVysion™ test was needed, as cytology is nearly 100% specific.^26^ If cytology was negative or atypical cells were found, the reflex UroVysion™ test was performed on the same urine or bladder washing specimen. The problem arose if FISH was positive and the subsequent cystoscopy was negative. The authors found that these were anticipatory positive cases because 50 to 80% patients with FISH positive test developed cancer within 29 months.

In one of the last published studies using UroVysion™ test, Kipp *et al.* have shown that also the percentage of polysomic cells (cells having an extra copy of one or more chromosomes) in the FISH positive patients is important.^27^ The result of more than 5% of abnormal cells correlated with the recurrence and the progression of urothelial carcinoma to muscle invasion in patients with non-(muscle)-invasive carcinoma. Furthermore, the result of more than 31% of abnormal cells was correlated to muscle invasion. However, a similar problem appeared as in previous studies, many patients with FISH positive test had negative cystoscopy, so the further treatment of these patients would have to be determined.

Obviously, as any diagnostic test also the UroVysion™ FISH test could give false positive results, namely signal splitting, few tetrasomic cells (cells of the G2M phase) or overlapping cells could be interpreted as polysomic cells.

On the other hand the test could also be false negative, specifically if there are no diagnostic cells in the sample or due to certain technical problems. As in other diagnostic tests, including cytology, it was also found to be negative in some low-grade urothelial tumours.

Nevertheless, there is a general agreement among cytopathologists that UroVysion™ FISH test is a new promising diagnostic tool in urinary cytology.^28^,^29^

### Experience of the Institute of Pathology, Faculty of Medicine, University of Ljubljana with UroVysion™ test

We started introducing UroVysion™ test by the end of 2008. The performance of the UroVysion™ test on a Papanicolaou stained slides of urine or bladder washings prepared by membrane filter imprint technique routinely used at our institute, was not yet reported.

**FIGURE 5.**
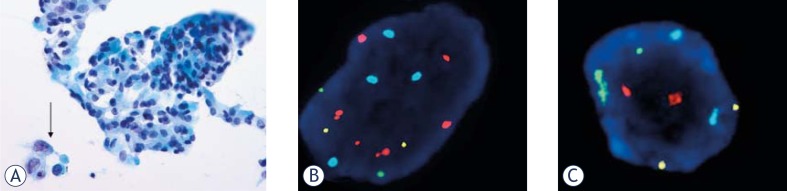
Mild cytological atypia of urothelial cells (arrow) in routine cytology bladder washing specimen (Papanicolaou, ×400) (A). Positive UroVysion test: 9 aneuploid cells (B). Majority were diploid cells (C).

Our approach was to find the area on the slide containing well preserved and well distributed atypical /representative cells which were marked by a diamond pencil for the subsequent testing by UroVysion™. UroVysion™ test was performed according to the manufacturer’s guidelines with two minor adjustments, the slides were first decolorized in acid ethanol and the enzyme digestion was lengthened to 28 minutes. Eighteen out of 29 tests were used to introduce and optimize a new method and further, eleven tests were used on diagnostic samples (Table 2). We found that all 5 cases of undetermined and suspicious atypia were UroVysion™ test positive, while also one case of mild cytological atypia that would be regarded as negative/benign was positive in a patient who was previously treated for non-invasive low-grade papillary carcinoma (Figure 5). As expected all 3 cases with positive/ malignant cytology were UroVysion™ test positive and one case with negative cytology was also negative on UroVysion™ test. We concluded that the cytopathological diagnosis could be improved in 6/7 (88%) of atypical-suspicious cases. However, further experience with the test will be needed and the correlation of the UroVysion™ test results to histopathological diagnosis on tissue biopsies is awaited in order to improve the diagnosis in increasing number of patients with urothelial carcinoma in Slovenia.^30^

Our initial impression is that the UroVysion™ test requires optimization to suit the procedures already used in one’s laboratory for the preparation of the fluid samples from the urinary tract. The introduction of UroVysion™ test requires initial staff training and additional equipment, foremost fluorescence microscope with appropriate filters. At the present the test is rather costly and time consuming.

## Conclusions

Malignant cytomorphological characteristics of exfoliated cells in urine or bladder washing can facilitate the diagnosis of primary or recurrent urothelial carcinoma, therefore the method remains a useful diagnostic test with high specificity. However, in cases with less pronounced cellular and nuclear atypia the cytopathological diagnosis is not reliable giving too many false negative results. Many ancillary tests were developed on urinary samples in the past two decades to overcome the low sensitivity of cytology for the detection of bladder cancer. The newest and most promising test is commercially available multicolour multitarget FISH UroVysion™ test which was introduced into routine diagnostics also at the Institute of Pathology, Faculty of Medicine, University of Ljubljana.

## Figures and Tables

**TABLE 1.** Cytological-histological correlation in 125 cases of urines and bladder washings with subsequent tissue biopsy from 2007 to 2009

**DIAGNOSIS**	**Cytology**	**Negative**	**Mild atypia**	Atypia NOS*	**Suspicious for carcinoma**	**Carcinoma**	**Total**
**Histology**							
LG** papillary urothelial carcinoma		5	4	8	9	2	28
HG*** papillary urothelial carcinoma		1	-	-	4	17	22
**Invasive urothelial carcinoma**		-	1	2	2	21	26
**Invasive and in situ urothelial carcinoma**		-	-	-	-	2	2
**In situ urothelial carcinoma**		-	-	1	3	7	11
**No malignancy**		21	4	2	8	1	36
**Total**		27	9	13	26	50	125

* NOS = not otherways specified;

** LG = low-grade;

*** HG = high-grade

**TABLE 2.** Results of the first set of the UroVysion™ test in patients with different cytopathological diagnoses on cell samples.

		**FISH UroVysion™ test**		
		**Negative**	**Positive**	**Total**
**Cytology**	No malignancy (negative)	1	-	1
	Mild Atypia	1	1	2
	Moderate atypia / suspicious for carcinoma	-	5	5
	Carcinoma (positive)	-	3	3
	Total	2	9	11

FISH = fluorescence *in situ* hybridization

## References

[1] Grunze H, Springss AI (1980). History of clinical cytology. A selection of documents.

[2] Koss LG, Koss LG, Melamed MR (2006). The lower urinary tract in the absence of cancer. Koss’s diagnostic cytology and its histopathologic bases.

[3] Grossfeld GD, Litwin MS, Wolf JS, Hirack H, Shuler CL, Agerter DC (2001). Evaluation of asymptomatic microscopic hematuria in adults: The American Urological Association best practice policy – part I: definition, detection, prevalence, and etiology. Urology.

[4] Grossfeld GD, Litwin MS, Wolf JS, Hirack H, Shuler CL, Agerter DC (2001). Evaluation of asymptomatic microscopic hematuria in adults: the American Urological Association best practice policy – part II: patient evaluation, cytology, voided markers, imaging, cystoscopy, nephrology evaluation, and follow-up. Urology.

[5] Koss LG, Koss LG, Melamed MR (2006). Tumors of the urinary tract in urine and brushings. Koss’s diagnostic cytology and its histopathologic bases.

[6] Rosenthal DL, Raab SS, Rosenthal DL (2005). Cytologic detection of urothelial lesions. Essentials in cytopathology series.

[7] http://www.thinprep.com/ [assessed July 1st, 2010]

[8] Lopes-Beltran A, Sauter G, Gasser T, Hartman A, Schmitz-Dräger BJ, Helpap B, Eble JN, Sauter G, Epstein JI, Sesterhenn IA (2004). Infiltrating urothelial carcinoma. WHO classification of tumours: pathology and genetics of tumours of the urinary system and male genital organs.

[9] Sauter G, Algaba F, Amin MB, Busch C, Cheville J, Gasser T, Eble JN, Sauter G, Epstein JI, Sesterhenn IA (2004). Non-invasive urothelial tumors. WHO classification of tumours: pathology and genetics of tumours of the urinary system and male genital organs.

[10] Bastacky S, Ibrahim S, Wilczynski SP, Murphy WM (1999). The accuracy of urinary cytology in daily practice. Cancer (Cancer Cytopathol).

[11] Curry JL, Wojcik EM (2002). The effect of the current World Health Organisation/International Society of Urologic Pathologists bladder neoplasm classification system on urine cytology results. Cancer.

[12] Greene LF, Hanash KA, Farrow GM (1973). Benign papilloma or papillary carcinoma of the bladder?. J Urol.

[13] Ross JS, Cohen MB (2000). Ancillary methods for the detection of reccurent urothelial neoplasia. Cancer.

[14] Us-Krašovec M (1998). Pretočna in slikovna citometrija: novi kvantitativni metodi. Onkologija.

[15] Böcking A, Striepecke E, Auer H, Füzesi L, Wied GL, Bartels PH, Rosenthal D, Schenk U (1994). Static DNA cytometry. Biological background, technique and diagnostic interpretation. Compendium on computerized cytology and histology laboratory.

[16] Tribukait B (1987). Flow cytometry in assessing the clinical agressiveness of genitourinary neoplasms. World J Urol.

[17] Sullivan PS, Nooraie F, Sanchez H, Hirschowitz S, Levin M, Rao PN (2009). Comparison of ImmunoCyt, UroVysion and urine cytology in detection of recurrent urothelial carcinoma: a “split-sample” study. Cancer Cytopathol.

[18] Tętu B (2009). Diagnosis of urothelial carcinoma from urine. Modern Pathol.

[19] Soloway MS, Briggman V, Carpinito GA, Chodak GW, Church PA, Lamm DL (1996). Use of a new tumor marker, urinary NMP22, in the detection of occult and rapidly recurring transitional cell carcinoma of the urinary tract following surgical treatment. J Urol.

[20] Nguyen CT, Jones JS (2008). Defining the role of NMP22 in bladder cancer surveillance. World J Urol.

[21] Richter J, Beffa L, Wagner U, Schraml P, Gasser TC, Moch H (1998). Patterns of chromosomal imbalances in advanced urinary bladder cancer detected by comparative genomic hybridization. Am J Pathol.

[22] Zhao J, Richter J, Wagner U, Roth B, Schraml P, Zellweger T (1999). Chromosomal imbalances in noninvasive papillary bladder neoplasms (pTa). Cancer Res.

[23] Sokolova IA, Halling KC, Jenkins RB, Burkhardt HM, Meyer RG, Seeling SA (2000). The development of a multitarget, multicolor fluorescence in situ hybridization assay for the detection of urothelial carcinoma in urine. J Mol Diag.

[24] http://www.urovysion.com/ [assessed July 1st, 2010]

[25] Bubendorf L, Grilli B, Sauter G, Mihatsch MJ, Gaser TC, Dalquen P (2001). Multiprobe FISH for enhanced detection of bladder cancer in voided urine specimens and bladder washings. Am J Clin Pathol.

[26] Yoder BJ, Skacel M, Hedgepeth R, Babineau D, Ulchaker JC, Liou LS (2007). Reflex UroVysion testing of bladder cancer surveillance patients with equivocal or negative cytology: a prospective study with focus on the natural history of anticipatory positive findings. Am J Clin Pathol.

[27] Kipp BR, Tanasescu M, Else TA, Bryant SC, Karnes RJ, Sebo TJ (2009). Quantitative fluorescent in situ hybridisation and its ability to predict bladder cancer recurrence and progression to muscle invasive bladder cancer. J Mol Diagn.

[28] Halling KC, Kipp BR (2007). Fluorescence in situ hybridization in diagnostic cytology. Hum Pathol.

[29] Schmitt FC, Longatto-Filho A, Valent A, Vielh P (2008). Molecular techniques in cytopathology practice. J Clin Pathol.

[30] Cancer Registry of Slovenia (2009). Cancer incidence in Slovenia 2006.

